# K_V_7 Channel Expression and Function Within Rat Mesenteric Endothelial Cells

**DOI:** 10.3389/fphys.2020.598779

**Published:** 2020-12-07

**Authors:** Samuel N. Baldwin, Shaun L. Sandow, Gema Mondéjar-Parreño, Jennifer B. Stott, Iain A. Greenwood

**Affiliations:** ^1^Vascular Biology Research Centre, Institute of Molecular and Clinical Sciences, St George’s University of London, London, United Kingdom; ^2^Biomedical Science, School of Health and Sports Science, University of the Sunshine Coast, Maroochydore, QLD, Australia; ^3^Department of Pharmacology and Toxicology, School of Medicine, Complutense University of Madrid, Madrid, Spain

**Keywords:** pharmacology, vascular biology, endothelial cell, K_V_7 channel, K_IR_ channel, carbachol

## Abstract

**Background and Purpose:** Arterial diameter is dictated by the contractile state of the vascular smooth muscle cells (VSMCs), which is modulated by direct and indirect inputs from endothelial cells (ECs). Modulators of KCNQ-encoded k_V_7 channels have considerable impact on arterial diameter and these channels are known to be expressed in VSMCs but not yet defined in ECs. However, expression of k_V_7 channels in ECs would add an extra level of vascular control. This study aims to characterize the expression and function of K_V_7 channels within rat mesenteric artery ECs.

**Experimental Approach:** In rat mesenteric artery, KCNQ transcript and K_V_7 channel protein expression were determined via RT-qPCR, immunocytochemistry, immunohistochemistry and immunoelectron microscopy. Wire myography was used to determine vascular reactivity.

**Key Results:** KCNQ transcript was identified in isolated ECs and VSMCs. K_V_7.1, K_V_7.4 and K_V_7.5 protein expression was determined in both isolated EC and VSMC and in whole vessels. Removal of ECs attenuated vasorelaxation to two structurally different K_V_7.2-5 activators S-1 and ML213. K_IR_2 blockers ML133, and BaCl_2_ also attenuated S-1 or ML213-mediated vasorelaxation in an endothelium-dependent process. K_V_7 inhibition attenuated receptor-dependent nitric oxide (NO)-mediated vasorelaxation to carbachol, but had no impact on relaxation to the NO donor, SNP.

**Conclusion and Implications:** In rat mesenteric artery ECs, K_V_7.4 and K_V_7.5 channels are expressed, functionally interact with endothelial K_IR_2.x channels and contribute to endogenous eNOS-mediated relaxation. This study identifies K_V_7 channels as novel functional channels within rat mesenteric ECs and suggests that these channels are involved in NO release from the endothelium of these vessels.

## Introduction

KCNQ-encoded K_V_7 channels are key regulators of arterial reactivity. Within the vasculature, of the five KCNQ subtypes, KCNQ4 is predominantly expressed, followed by KCNQ5 > KCNQ1; with little to no contribution from KCNQ2/3 (Ohya et al., 2003; Yeung et al., 2007; Ng et al., 2011). In human and rodent blood vessels K_V_7 channels contribute to resting tone (Ohya et al., 2003; Yeung et al., 2007; Mackie et al., 2008; Ng et al., 2011), whereby their blockers such as linopirdine or XE991 produce contractions or enhance vasoconstrictor responses. In addition, a range of K_V_7 channel activators including retigabine, S-1 and ML213 are effective relaxants of pre-contracted arterial tone. Furthermore, K_V_7 channels also represent functional end targets for a myriad of endogenous vasoactive responses, wherein channel activity is impaired during PKC-mediated vasoconstriction (Brueggemann et al., 2006) and enhanced as a result of cGMP and cAMP dependent receptor-mediated vasodilation (e.g., Chadha et al., 2012; Khanamiri et al., 2013; Stott et al., 2014, 2015; Mani et al., 2016; Brueggemann et al., 2018; Mondéjar-Parreño et al., 2019). To date, vascular K_V_7 channel studies have focused predominantly on vascular smooth muscle cells (VSMCs) or whole arteries, and as a result it is currently unclear whether endothelial cells (ECs) express K_V_7 channels and if so, what their functional role may be.

Endothelial cells form the inner layer of blood vessels and constitute a paracrine signaling platform which regulates VSMC contractility, vascular resistance and ultimately blood flow through the release of nitric oxide (NO), prostacyclin, epoxyeicosatrienoic acid and others; including the generation and spread of endothelium-derived hyperpolarization (EDH) (McGuire et al., 2001). Myoendothelial (ME) projections within fenestrations (holes) of the internal elastic lamina (IEL) facilitate the presence gap junctions (MEGJs) at a proportion of such sites (∼50% in adult rat 1st–3rd order ‘large’ mesenteric arteries; MA; Sandow et al., 2009) which permits heterocellular electrochemical coupling via connexin proteins (Sandow et al., 2012). These sites enable the transfer of EC-derived signals via the flow of both small molecules < ∼1 kDa and current between the cells. In endothelium-dependent relaxation of rat MA, a fundamental role for small/intermediate conductance calcium-activated potassium (SK_Ca_ and IK_Ca_, respectively; Sandow et al., 2006; Dora et al., 2008), transient receptor potential canonical type 3 (Senadheera et al., 2012), inwardly rectifying potassium channels (K_IR_2) (Goto et al., 2004), as well as inositol-1,3,4 trisphosphate receptor/s (Fukao et al., 1997) has been demonstrated.

Identification of K_V_7 expression in ECs would open up a new layer of vascular control by these channels and is a necessary requisite for understanding the role of these channels in vascular health and disease. This study aims to ascertain whether rat mesenteric ECs express K_V_7 channels, and if so, what their functional implications may be. This study shows that K_V_7 channels are expressed in rat mesenteric artery ECs and contribute to both K_V_7 activator-mediated relaxation via a potential functional interaction with K_IR_2 channels and endothelial NO synthase (eNOS)-dependent axis of carbachol (CCh)-mediated vasorelaxation.

## Materials and Methods

### Animal Models

Experiments were performed on MA from Male Wistar rats (Charles River, Margate, United Kingdom) ages 11–14 weeks (200–350 g) from the Biological Research Facility, St George’s, London, United Kingdom; and from the Animal Resources Center, Perth, Australia. Animals were housed in cages with free access to water and food (RM1; Dietex Inter-national, United Kingdom) *ad libitum*, with a 12-h light/dark cycle and constant temperature and humidity (21 ± 1°C; 50% ± 10% humidity) in accordance with the Animal (Scientific Procedures) Act 1986, the guidelines of the National Health and Medical Research Council of Australia and the UNSW Animal Ethics and Experimentation Committee (AEEC #18/86B). Animals were kept in LSB Aspen woodchip bedding. Animals were culled by either cervical dislocation with secondary confirmation via cessation of the circulation by femoral artery severance or were anesthetized with sodium pentathol (intraperitoneal, 100 mg/kg) in accordance with Schedule 1 of the ASPA 1986.

Either whole mesenteric plexus or 2nd/3rd/4th order MA were used with vessel order identified from the second bifurcation of the superior MA. Arteries were dissected, cleaned of fat and adherent tissue and stored on ice within physiological salt solution (PSS) of the following composition (mmol-L^–1^); 119 NaCl, 4.5 KCl, 1.17 MgSO_4_.7H_2_0, 1.18 NaH_2_PO_4_, 25 NaHCO_3_, 5 glucose, 1.25 CaCl_2_.

### Reverse Transcription Quantitative Polymerase Chain Reaction

Second and third order MA segments were enzymatically digested to obtain either freshly isolated ECs (as previous, Greenberg et al., 2016) or VSMCs. Briefly, vessels were washed in Hanks’ Balanced Salt Solution (HBSS; ThermoFisher Scientific, GIBCO, 14170-088) containing 50 μmol-L^–1^ CaCl_2_ for 5 min at 37°C, the media was then replaced in HBSS containing 50 μmol-L^–1^ CaCl_2_ with 1 mg/mL collagenase IA (Sigma Aldrich, C9891, United Kingdom) for 15 min at 37°C. Vessels were washed in HBSS containing 50 μmol-L^–1^ CaCl_2_ for 10 min at 37°C. The supernatant was removed and the vessels suspended in fresh HBSS containing 75 μmol-L^–1^ CaCl_2_. For RT-qPCR, ECs were dissociated using a wide-bore smooth-tipped pipette and identified under the microscope (x10) as sheets of cells independent from the vessel which were harvested and stored separately from the residual VSMCs.

mRNA from both isolated ECs and VSMCs was extracted using Monarch Total RNA Miniprep Kit (New England BioLabs, Ipswich, MA, United States) and reverse transcribed via LunaScript RT SuperMix Kit (New England BioLabs, Ipswich, MA, United States). Quantitative analysis of relative gene expression was assessed via CFX-96 Real-Time PCR Detection System (BioRad, Hertfordshire, United Kingdom). Samples were run in duplicate to account for variation. Samples were run in BrightWhite qPCR plate (Primer Design, Camberley, United Kingdom), with each well containing 20 μL of reaction solution containing: 10 μL of PrecisionPLUS qPCR Master Mix (Primer Design, Camberley, United Kingdom), 300 nmol-L^–1^ of gene specific target primer (Thermofisher scientific, Waltham, MA, United States) and 10 ng of cDNA sample made up to 20 μL total volume with nuclease free water. Run protocol: (1) activation step (15 min:95°C), (2) denaturation step (15 s: 94°C), (3) annealing step (30 s: 55°C), and (4) extension step (30 s: 70°C). Steps 2- 4 were repeated x 40. Quantification cycle (Cq) was determined via Bio-Rad CFX96 Manager 3.0. Cq values were normalised to housekeeper genes expressed as a 2^–ΔCq^ when compared to appropriate reference genes including 14-3-3 Zeta (YWHAZ) and glyceraldehyde-3-phosphate dehydrogenase (GAPD). Cell isolation for VSMCs and ECs was validated by either positive expression of VSMC specific marker α-actin 2 (*Acta2)* or EC specific marker-platelet endothelial cell adhesion molecule-1 *(Pecam-1)* respectively. See Table 1 for a list of the primers used in the following (Jepps et al., 2011; Askew Page et al., 2019; ThermoFisher Scientific).

**TABLE 1:** RT-qPCR primer sequences.

**Gene**	**(+) Forward primer sequence**	**Gene accession number**	**Amplicon (bp)**
	**(−) Reverse primer sequence**		
*Acta2*	ATCCGATAGAACACGGCATC	NM_031004.2	228
	AGGCATAGAGGGACAGCACA		
*Pecam1*	CTCCTAAGAGCAAAGAGCAACTTC	NM_031591.1	100
	TACACTGGTATTCCATGTCTCTGG		
*Kcnq1*	TGGGTCTCATCTTCTCCTCC	NM_032073	124
	GTAGCCAATGGTGGTGACTG		
*Kcnq2*	AAGAGCAGCATCGGCAAAAA	NM_133322	101
	GGTGCGTGAGAGGTTAGTAGCA-		
*Kcnq3*	CAGCAAAGAACTCATCACCG	AF091247	161
	ATGGTGGCCAGTGTGATCAG		
*Kcnq4*	GAATGAGCAGCTCCCAGAAG	XM_233477.8	133
	AAGCTCCAGCTTTTCTGCAC		
*Kcnq5*	AACTGATGAGGAGGTCGGTG	XM_001071249.3	120
	GATGACCGTGACCTTCCAGT		

### Immunocytochemistry

Freshly dispersed ECs (as above), together with residual VSMCs were left for 1hr before use. Cells were then fixed in 4% paraformaldehyde (Sigma-Aldrich, United Kingdom) in PBS for 20 min at RT as previously described (Barrese et al., 2018a). Cells were treated with 0.1 mol-L^–1^ glycine for 5 min and incubated for 1 h with blocking solution (PBS-0.1% Triton X-100-10% bovine serum albumin) at RT. Following incubation overnight at 4°C with primary antibodies (Table 2) diluted in blocking solution (anti-PECAM-1 for ECs, anti-α-actin for VSMCs and anti-K_V_7.1, K_V_7.4, and K_V_7.5 channel for ECs/VSMCs), cells were then washed for 20 min with PBS, incubated for 1 h at RT with the secondary conjugated antibodies diluted in blocking solution. Excess secondary antibody was removed by washing with PBS and cells mounted using media containing 4′,6-diamidino-2-phenylindole (DAPI) for nuclear counterstaining. Using triple staining, ECs and VSMC were differentiated via the following: ECs were positive for anti PECAM-1 and negative for anti-α-actin; while VSMC was positive for anti-α-actin and negative for anti-PECAM-1 (data not shown). Cells were analyzed using a Zeiss LSM 510 Meta argon/krypton laser scanning confocal microscope (Image Resource Facility, St George’s University, London).

**TABLE 2:** Immunocyto/histochemistry reagents and use (Jepps et al., 2009; Chadha et al., 2012).

**Reagent purpose**	**Detail**	**Source**	**Predicted MW, kDa**	**Epitope**	**[used]**	**Peptide availability**	**Raised in**
*Primary antibodies*	Kv7.1/KCNQ1	Pineda Antikörper-Service, Germany	75	N-terminus	1:100	No	Rabbit
	Kv7.4/KCNQ4	NeuroMab, cat no 75-082, 1 mg/ml	77	Hu aa 2-77, clone N4/36 IgG	1:200 (5 μg/ml)	No	Mouse
	Kv7.4/KCNQ4*	Abcam, ab65797, lot GR94754, whole serum	77	N’ domain	1:100 not available	No	Rabbit
	Kv7.5/KCNQ5*	Millipore ABN1372-q2476155; 1 ml/ml	∼103	Human IgG	1:100 (10 μg/ml)	No	Rabbit
	PECAM-1/CD31	Santa Cruz Biotechnology, Sc-1506, 200 μg/ml	130	699–727 aa at the C-terminus	1:100 (2 μg/ml)	–	Goat
	SM-α-actin	Sigma Aldrich A2547	∼42	N-terminal	(1:100) not available	–	Mouse
*Nuclear labels/cell patency markers*	DAPI, Vectasheild	Vectorlabs	–	Nucleic acid		–	–
	propidium iodide (PI)	Sigma, P4170	–	Nucleic acid	10 nM	–	–
*Immuno-histochemistry secondary antibodies*	Mouse 568	Abcam, ab175700, lot GR320062-4, 2 mg/ml	–	IgG	1:100 (20 μg/ml)	–	Donkey
	Rabbit 546	Thermofisher, A-11035	–	IgG	1:100 (20 μg/ml)	–	Goat
	Rabbit 633	Merck, SAB4600132, lot 15C0423, 2 mg/ml	–	IgG	1:100 (20 μg/ml)	–	Donkey
*Immuno-cytochemistry secondary antibodies*	Mouse 488	Thermofisher, A21202, 2 mg/mL	–	IgG	1:100 (0.02 mg/ml)	–	Donkey
	Rabbit 568	Thermofisher, A10042, 2 mg/mL	–	IgG	1:100 (0.02 mg/ml)	–	Donkey
	Goat 633	Thermofisher, A21082, 2 mg/mL	–	IgG	1:100 (0.02 mg/ml)	–	Donkey
*Isotype controls*	Mouse IgG	ThermoFisher, 10400C	–	IgG	5 mg/ml	–	Mouse
	Rabbit IgG	ThermoFisher, S31235	–	IgG	10 mg/ml	–	Rabbit
*Immunoelectron microscopy secondary antibodies*	5 nm Au anti- rabbit	Merck, G7277, lot SLB3882V	–	IgG	1:100	–	Goat
	10 nm Au anti- rabbit	Merck, G7402	–	IgG	1:100	–	Goat

*CD31, cluster of differentiation 31; DAPI, 4’,6-diamidino-2-phenylindole; PECAM, platelet endothelial cell adhesion molecule.*

### Cell Culture

Chinese Hamster Ovary (CHO) cells were maintained in DMEM supplemented with 10% fetal bovine serum, 2 mmol-L^–1^ L-glutamine, and 1% penicillin/streptomycin (Sigma Aldrich, Dorset, United Kingdom) and maintained at 37°C with 5% CO_2_ in an incubator. Cells were plated in a 24-well plate, incubated for 24hr then transfected with either K_V_7.1, K_V_7.4 or K_V_7.5 plasmids using Lipofecamine 2000 (Thermofisher, Paisley, United Kingdom) as described previously (Barrese et al., 2018a). After 24 h, cells were fixed and stained as described above.

### Immunohistochemistry

Animals were anesthetized with sodium pentathol (intraperitoneal, 100 mg/kg) and perfusion fixed (Sandow et al., 2004) in 2% paraformaldehyde in 0.1 mol-L^–1^ PBS. Third to 4th order MA segments were dissected, opened laterally and pinned as a sheet to a Sylgard dish. Segments were washed in PBS (3 × 5 min), incubated in blocking buffer (PBS with 1% BSA and 0.2% Triton) at room temperature (RT) for 2 h and then overnight with primary antibody (Table 2) in blocking buffer at 4°C, washed again (3 × 5 min with gentle agitation), and incubated in secondary antibody (Table 2; matched to the respective primary) in PBS with 0.1% Triton in PBS for 2 h at RT. Tissue was mounted on slides in anti-fade media containing propidium iodide (PI) or DAPI (Table 2) and imaged with uniform confocal settings. Incubation of tissue with secondary only was used as a ‘zero’ setting for confocal imaging. Controls involved substitution of primary with isotype control, with concentration (where provided by manufacturer) matched, or 10-fold higher than the respective antibody of interest (Table 2). Working Ab dilutions were prepared in accordance with previous work (Jepps et al., 2009; Chadha et al., 2012). Confocal image stacks were collected at 0.2 μm intervals. The optimal rinsing protocol was determined by incubating in secondary only; and rinsing after successive 5 min incubations until fluorescence was reduced to background. Note that if this was not done secondary alone was specifically and highly localized to IEL hole sites; as potential false positives at such sites; suggesting that such sites have an affinity for IgG-secondary label alone.

### Electron Microscopy

Animals were anesthetized as above and perfusion fixed in 0.2% glutaraldehyde and 2% paraformaldehyde in 0.1 mol-L^–1^ PBS (pH 7.4). MA segments (∼2 mm in length) were washed (3 × 5 min) and processed in a Leica EMPACT 2 high-pressure freezer using 0.7% low melting agarose as a cryoprotectant. Samples were then freeze-substituted in a Leica AFS2 into 0.2% uranyl acetate in 95% acetone (from −85 to −50°C) and infiltrated with Lowicryl (at −50°C), before UV polymerization (2 days each at −50 and 20°C; Zechariah et al., 2020). Conventional transmission electron microscopy (TEM) was conducted using standard procedures (Sandow et al., 2002, 2004).

Individual serial transverse sections (∼100 nm) were mounted on Formvar-coated slot grids and processed for antigen localization as for confocal immunohistochemistry (per above and Table 2). The secondary used was 5 or 10 nmol-L^–1^ colloidal gold-conjugated antibody (1:40; 2 h) in 0.01% Tween-20. Sections were imaged at x10-40,000 on a JEOL transmission electron microscope at 16 MP (Emsis, Morada G3). Background gold label density was determined from randomly selected (4x) 1 × 1 μm regions per sample of lumen and IEL, compared to the same sized regions of interest in EC profiles.

### Wire Myography

Second order MA segments (∼2 mm in length) were mounted on 40 μm diameter tungsten wire in a tension myograph chamber (Danish Myo Technology, Arhus, Denmark) containing 5 mL of PSS (composition, as above) oxygenated with 95% O_2_ and 5% CO_2_ at 37°C. Vessels then underwent a passive force normalization process to achieve an internal luminal circumference at a transmural pressure of 100 mmHg (13.3 kPa) to standardize pre-experimental conditions (Mulvany and Halpern, 1976). Force generated was first amplified by a PowerLab (ADInstruments, Oxford, United Kingdom), and recorded by LabChart software (ADInstruments, Oxford, United Kingdom). Vessels were then challenged with 60 mmol-L^–1^ [K^+^] to determine viability, and then constricted with 10 μmol-L^–1^ methoxamine (MO), an α-1 adrenoreceptor agonist, EC integrity was then determined via addition of 10 μmol-L^–1^ carbachol (CCh), a synthetic acetylcholine analog. Vessels displaying ≥ 90% vasorelaxation in response to CCh were considered EC positive (EC+). Vessels were denuded of ECs by gently passing a human hair through the lumen. Vessels expressing ≤ 10% vasorelaxation in response to CCh were considered EC negative (EC−). During functional investigations, all vessels were pre-constricted with the thromboxane A2 receptor agonist U46619 (300 nmol-L^–1^) to elicit an EC_80_ contraction. Concentration-dependent relaxant responses to S-1 (0.1–10 μmol-L^–1^), ML213 (0.1–10 μmol-L^–1^), ML277 (0.03–10 μmol-L^−1^), CCh (0.3–10 μmol-L^–1^) and *S*-nitroprusside (SNP; 0.01–3 μmol-L^–1^) were determined in the presence and absence of ECs, linopirdine (10 μmol-L^–1^), HMR-1556 (10 μmol-L^–1^), carbenoxolone (100 μmol-L^–1^), ML133 (20 μmol-L^–1^), barium chloride (BaCl_2_; 100 μmol-L^–1^), L-nitroarginine methyl ester (L-NAME; 100 μmol-L^–1^), TRAM34 (1 μmol-L^–1^), Apamin (10 nmol-L^–1^), 4-aminopyridine (4-AP; 1 mmol-L^–1^), and tetraethylamonium (TEA; 1 mmol-L^–1^).

### Data and Statistical Analysis

All functional figures express mean data from at least 5 animals ± standard error of the mean (SEM). Experiments comparing groups of unequal numbers are present due to technical failure or expiry of tissue during isometric tension recording. For functional experiments involving cumulative concentrations, a transformed data set was generated using; X = Log(X), to reduce representative skew. A four parametric linear regression analysis was then performed using the following equation; [Log(Agonist) vs. response – variable slope (four parameters bottom/hillslope/top/EC_50_)] using GraphPad Prism (Version 8.2.0) to fit a concentration effect curve (CEC) to the figure. For data comparing multiple groups, a two way-ANOVA followed by a *post hoc* Bonferonni test in order to account for type 1 errors in multiple comparisons was performed for comparison of mean values. For data comparing two groups, an unpaired parametric T-test was was performed. Significance values are represented as follows; *P* < 0.05 (^∗^). *n* = (x), number of animals used. *N* = (x), number of segments used. The data and statistical analysis comply with the recommendations on experimental design and analysis in pharmacology (Curtis et al., 2018).

## Results

### Identification of K_V_7 Channels Within MA ECs

**FIGURE 1:**
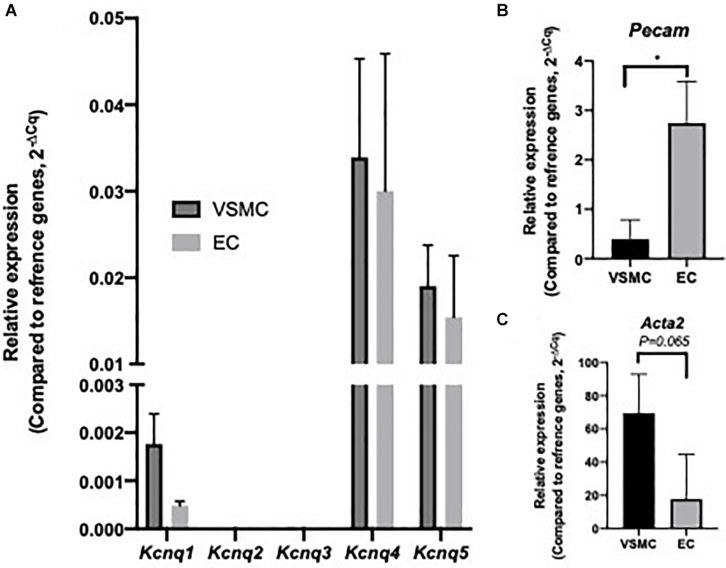
Expression profile of *Kcnq* genes within isolated rat mesenteric vascular smooth muscle cells (VSMCs) and endothelial cells (ECs). RT-qPCR analysis of relative expression of *Kcnq1-5* in isolated VSMCs and ECs **(A)**. Cell specific markers *Acta2* and *Pecam*, for VSMCs and ECs respectively, were measured to determine sample purity **(B,C)**. Values expressed as mean ± SEM of (2^–ΔCq^) of ΔCq values generated from appropriate housekeeper genes (*n* = 3). Statistical significance is defined as *P < 0.05 **(B,C)**.

Initial investigation sought to identify *Kcnq/*K_V_7 transcript and protein within MA ECs. Transcript levels for *Kcnq1-5* and EC and VSMC markers were determined in cell lysates from isolated MA VSMC and EC (per Methods). Both ECs and VSMCs expressed *Kcnq4* > *Kcnq5* > *Kcnq1* with no expression of *Kcnq2/3* (Figure 1A) similar to previous studies (Ohya et al., 2003; Yeung et al., 2007; Ng et al., 2011). However, a comparative reduction in the relative abundance of *Kcnq1* was observed in MA ECs when compared to VSMCs (Figure 1A). Cell isolation efficiency is demonstrated by a reduction in EC marker *Pecam* within VSMCs cell lysates when compared to ECs (*P* ≤ 0.05) and a reduction in VSMCs marker *Acta2* in EC cell lysates when compared to VSMCs (*P* = 0.065, Figures 1B,C).

**FIGURE 2:**
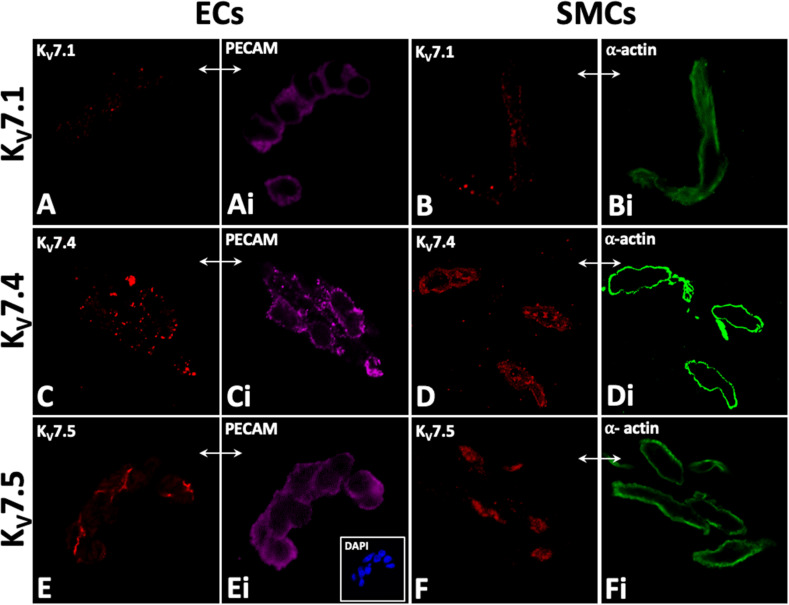
Immunocytochemistry demonstrates K_V_7.1, K_V_7.4, and 7_V_.5 localization in rat mesenteric artery isolated endothelial (EC; **A,C,E**) and smooth muscle cells (SMC; **B,D,F**). Punctate labelling for Kv7.1, Kv7.4, and 7.5 was present in isolated EC and SMCs (**A,C,E** and **B,D,F**). PECAM and smooth muscle a-actin were used as markers for isolated endothelial and smooth muscle cells, respectively (**Ai,Ci,Ei** and **Bi,Di,Fi** respectively); e.g. comparative-same cells, double arrows, **A–Fi**). 4′,6-diamidino-2-phenylindole (DAPI; e.g. inset, **C**) were used as nuclear markers to verify cell patency. *n* = 4–6, representative of different source tissue **(A–Fi)**. Double arrows from insets indicate same panel as main, but to different marker.

**FIGURE 3:**
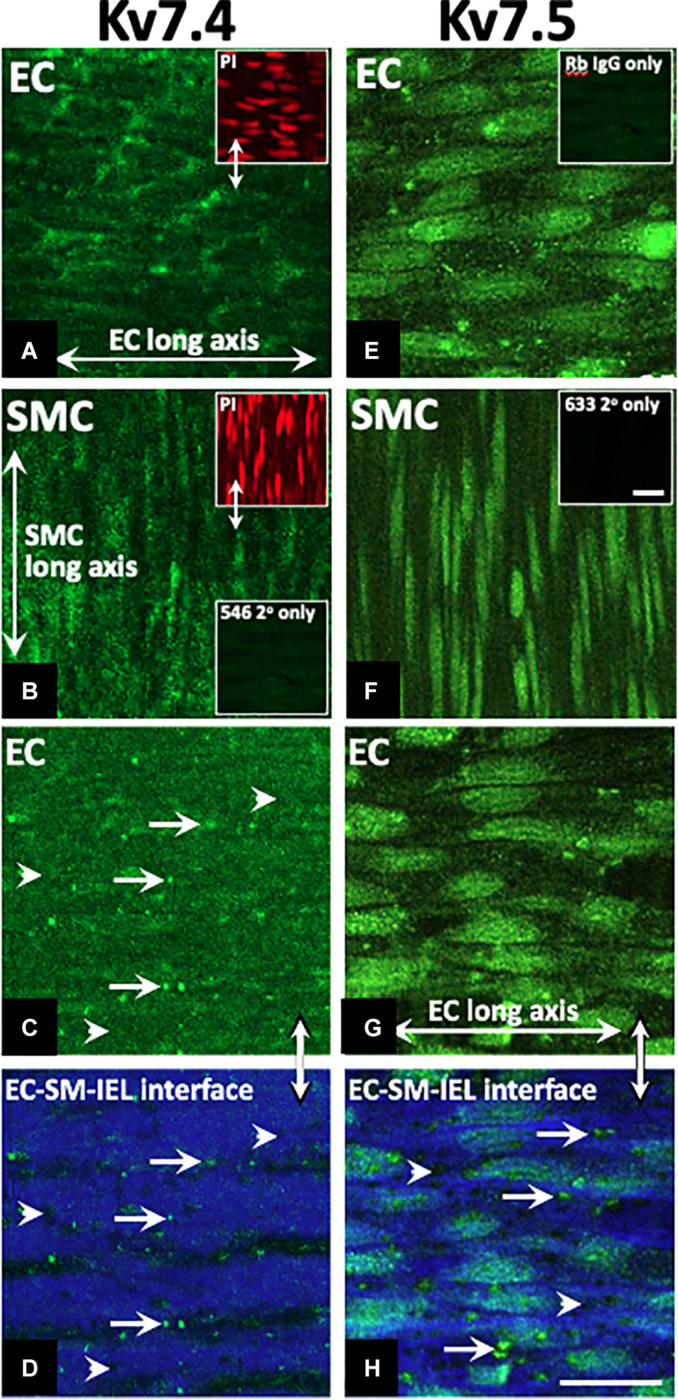
Immunohistochemistry demonstrates Kv7.4 and 7.5 localization in rat mesenteric artery endothelium (EC) and smooth muscle cells (SMCs) with a focus on internal elastic lamina (IEL) hole sites in whole mount tissue. Whole mounts of confocal stacks through the EC and SMC of K_v_7.4 (**A,B**, respectively) and Kv7.5 (**E,F**, respectively). Confocal image stacks through the luminal EC to SM border **(C,G)** show diffuse/punctate K_v_7.4/K_V_7.5 across the cell membrane with some punctate higher-level fluorescence at localized sites (e.g. full arrows; **C**). When single images were taken at the EC-IEL-SM interface **(D,H)**, punctate higher-level fluorescence densities were present (e.g. **D,H** arrows; **H**, higher magnification inset) at *a proportion* of IEL hole sites (e.g., **D,H** arrowheads). Propidium iodide (PI; e.g., upper left inset, **A,B**) were used as nuclear markers to verify cell and layer patency. Incubation in secondary only separately for each of the 3 fluorophores (Table 2; e.g. lower left inset, **B**; inset, **F**) were used to set ‘zero’ confocal settings. Incubation in isotype IgG control only (e.g., **E**, inset) showed fluorescence equivalent to secondary alone (e.g., **B**, lower inset; **F**, inset). *n* = 4, representative of different source. Longitudinal vessel axis, left to right. Double arrows indicate same site, but at different focal planes **(C,D,G,H)**, and location of inset **(D)**. Bar, 25 μm.

K_V_7.1, K_V_7.4, and K_V_7.5 were detected in isolated ECs by immunodetection (Figures 2A,C,E). K_V_7.4 had a punctate distribution in isolated ECs (Figure 2C) whereas K_V_7.5 label appeared to be predominantly cytoplasmic with some diffuse label around the nucleus (Figure 2E). Similar to previous reports (Zhong et al., 2010; Oliveras et al., 2014; Mills et al., 2015; Morales-Cano et al., 2015; Barrese et al., 2018a). K_V_7.1, K_V_7.4, and 7.5 were also identified in isolated MA VSMCs (Figures 2B,D,F). K_v_7.1 staining in ECs was negligible compared to VMSCs (Figures 2A,B). ECs and VSMCs were identified by positive expression of either PECAM (Figures 2Ai,Ci,Ei) or α-actin (Figures 2Bi,Di,Fi) respectively. Antibody specificity was determined via positive staining in CHO cells transfected with purported target and negative staining in non-transfected control cells (Supplementary Figure 1). Importantly, as ion channel expression can alter in isolated cells K_V_7.4 and K_V_7.5 were also detected in both ECs and VSMCs in *en face* whole-mount arteries (Figures 3A,B,E,F). Notably, both K_V_7.4 and 7.5 were expressed at a proportion of IEL hole sites at an apparently higher level than the associated EC membrane label (Figures 3C,D,G,H). We also detected K_V_7.4 and K_V_7.5 in EC by immuno-gold electron microscopy (Supplementary Figure 2). These studies identify K_V_7.4 and K_V_7.5 in ECs as well as smooth muscle cells.

### Removal of ECs Modulates K_V_7.2-5 Activator Efficacy

**FIGURE 4:**
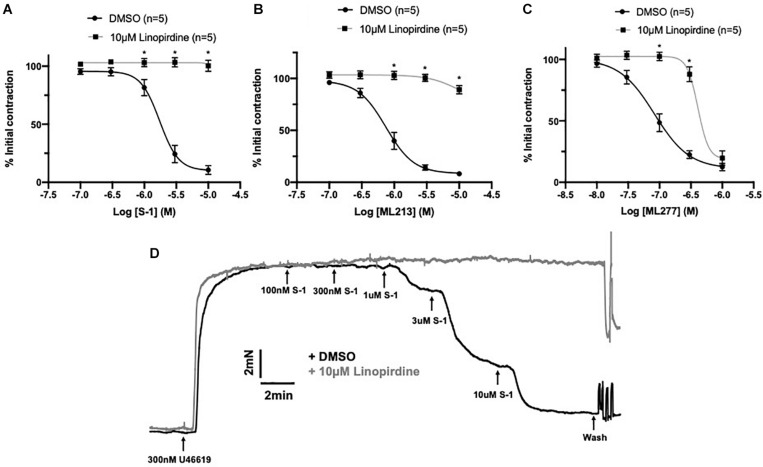
Sensitivity of Kv7 activator relaxations to 10 μmol-L^–1^ linopirdine. In vessels pre-incubated in DMSO solvent control, K_V_7.2-5 channel activators S-1 and ML213 relax pre-constricted arterial tone (300 nmol-L^–1^ U46619; black) and are ablated by pre-incubation with pan K_V_7 channel inhibitor linopirdine (10 μmol-L^–1^; gray; *n* = 5; **A,B**). In vessels pre-incubated in DMSO solvent control, K_V_7.1 channel activator ML277 relaxes pre-constricted arterial tone (300 mol-L^–1^ U46619; black) and is ablated by linopirdine (10 μmol-L^–1^; gray) to a threshold of 300 nmol-L^–1^ ML277 (*n* = 5; **C**). A representative trace of S-1-mediated vasorelaxation ± 10 μmol-L^–1^ linopirdine **(D)**. All values are expressed as mean + SEM. A two-way statistical ANOVA with a *post hoc* Bonferroni test was used to generate significance values. Statistical significance is defined as *P < 0.05 **(A–C)**.

A comprehensive pharmacological analysis was undertaken to determine if K_V_7 channels have a functional role in MA ECs via a reductive approach. Initially, the effects of K_V_7 channel modulators were examined in endothelium intact or denuded MAs. The structurally dissimilar K_V_7.2–7.5 activators S-1 and ML213 interact with the same pharmacophore centered around a tryptophan in the S5 domain (Schenzer, 2005; Bentzen et al., 2006; Brueggemann et al., 2014; Jepps et al., 2014). ML277 is a potent activator of K_V_7.1 (Yu et al., 2013) with a 100-fold increase in selectivity for K_V_7.1 compared to K_V_7.2-5 (Yu et al., 2013). Consistent with previous findings (Chadha et al., 2012; Jepps et al., 2014), S-1- and ML213-mediated vasorelaxation was ablated by pre-incubation with 10 μmol-L^–1^ of the pan-K_V_7 channel inhibitor linopirdine (Schnee and Brown, 1998; Figures 4A,B,D). Relaxation produced by 10–300 nmol-L^–1^ ML277 were also prevented by pre-incubation with linopirdine (Figure 4C). However, relaxation produced by concentrations > 1 μmol-L^–1^ ML277 was not attenuated by linopirdine and are therefore not mediated by K_V_7.1 activation.

**FIGURE 5:**
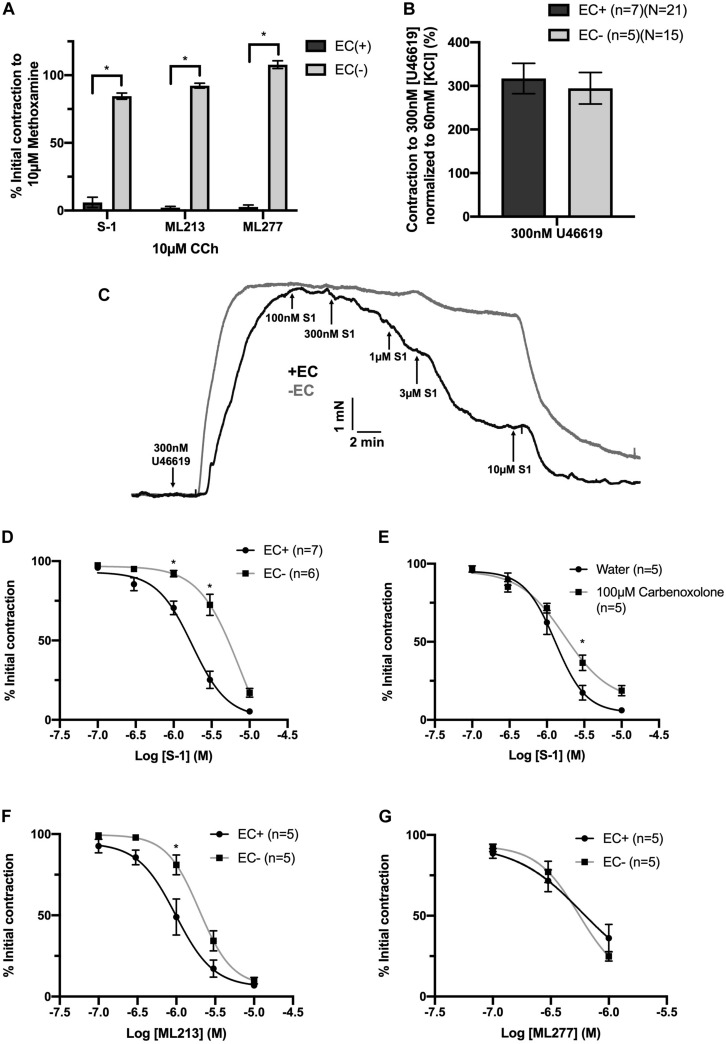
K_V_7.2-5 channel activator mediated vasorelaxation was significantly attenuated by EC removal. Removal of ECs ablated vasorelaxation in response to 10 μmol-L^–1^ CCh following pre-constriction with 10 μmol-L^–1^ MO **(A)**. Removal of ECs (gray) has no effect on vasoconstriction to U46619 (300 nmol-L^–1^) when normalized to vasoconstriction to KCl (60 mmol-L^–1^; *n* = 5–7; *n* = 15-21; **B**). Representative trace of EC S-1-mediated vasorelaxation in EC(–) denuded vessels (gray) vs. EC(+) vessels (black; **C**). S-1-mediated vasorelaxation was significantly attenuated by EC removal and 100 μmolL^–1^ carbenoxolone (gray; *n* = 5–7; **D,E**). ML213 but not ML277-mediated vasorelaxation was significantly attenuated by EC removal (gray; *n* = 5–7; **D,E**). Removal of ECs (gray) has no effect on ML277 within its range of linopirdine sensitivity (*n* = 5; **F**). All values are expressed as mean + SEM **(A–G)**. A two-way statistical ANOVA with a *post hoc* Bonferroni test was used to generate significance values. Statistical significance is defined as *P < 0.05 **(A–G)**.

Endothelial removal for the following experiments was confirmed by ablation of vasorelaxation in response to 10 μmol-L^–1^ CCh (Figure 5A). Endothelium denudation by mechanical abrasion has no impact on the peak contraction produced by 300 nmol-L^–1^ U46619 (Figure 5B), but significantly attenuated the potency of S-1 mediated vasorelaxation increasing EC_50_ from 2 ± 0.2 μmol-L^–1^ to 3 ± 0.7 μmol-L^–1^ (Figures 5C,D). S-1 mediated relaxation was also impaired via the non-selective gap junction inhibitor carbenoxolone (Tare et al., 2002) (water *E*_max_ = 6.11 ± 1.82% vs. carbenoxolone *E*_max_ = 18.7 ± 3.25%; Figure 5E). In addition, the potency of ML213 was also impaired by endothelial removal (EC(+) EC_50_ = 1 ± 0.2 μmol-L^–1^ vs. EC(−) EC_50_ = 3 ± 0.2 μmol-L^–1^; Figure 5F) in a fashion analogous to S-1. However, the linopirdine-sensitive relaxation produced by ML277 was not affected by endothelial removal (Figure 5G).

### EC K_IR_ Channels Modulate K_V_7.2-5 Activator Sensitivity

Having identified that the presence of the endothelium modulates responses to K_V_7 activators, experiments were performed to identify the underlying mechanism/s involved. *Murine* endothelial KCNJ2-encoded K_IR_2.1 channels have been identified as ‘signal boosters’ that enhance EC-derived relaxation (Sonkusare et al., 2016). Comparatively, the current literature regarding *rat* mesenteric K_IR_2 channels is limited. In brief, rat MA endothelium expresses K_IR_2.1 (Dora et al., 2008), inwardly rectifying Ba^2+^ sensitive channels are restricted to the endothelial layer (Crane et al., 2003a) and K_IR_ channels contribute to acetylcholine-mediated responses (Goto et al., 2004). We propose that like mice, rat mesenteric ECs express functional K_IR_2 channels that propagate EC signals in a similar process. Therefore, we performed a series of studies investigating the effect of two characterized K_IR_2 blockers, BaCl_2_ (Hagiwara et al., 1978) and ML133 (Wang et al., 2011) on K_V_7 activator-mediated vasorelaxation.

**FIGURE 6:**
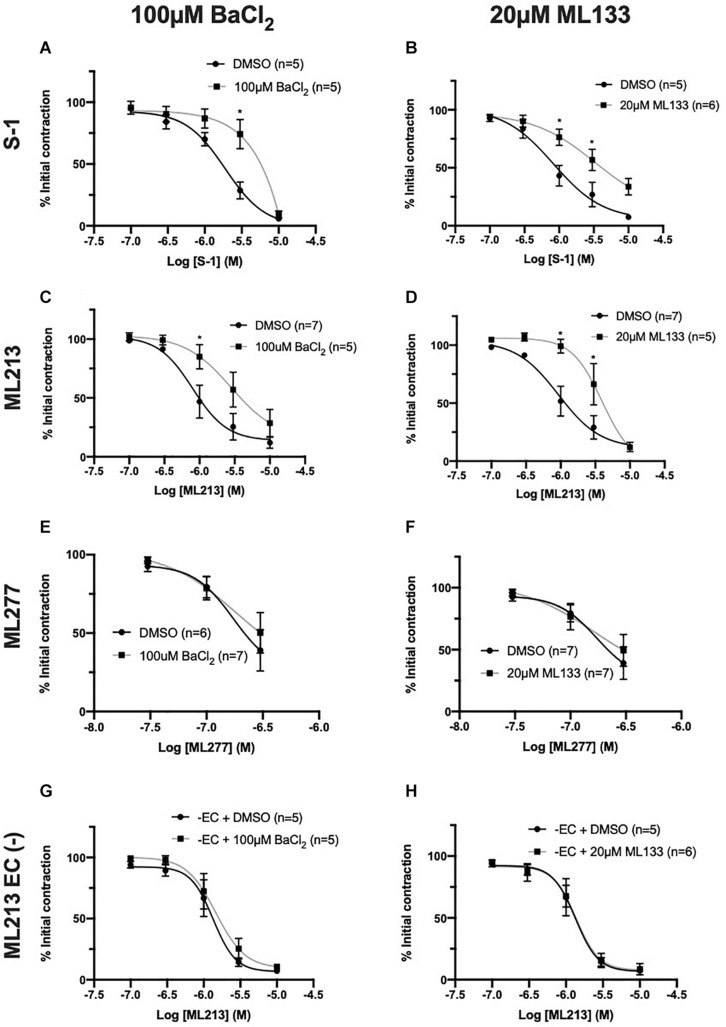
K_V_7.2-5 channel activator-mediated vasorelaxation was attenuated by two structurally different K_IR_ channel blockers, BaCl_2_ and ML133, but not In EC denuded vessels. K_V_7.2-5 channel activator S-1 and ML213 effects were significantly attenuated by pre-incubation with the K_IR_2 channel inhibitor BaCl_2_ (100 μmol-L^–1^; black; *n* = 5–7; **A,C**). K_V_7.2-5 channel activator S-1 and ML213 effects were significantly attenuated by pre-incubation with a selective K_Ir_ 2.1 channel inhibitor, ML133 (20 μmol-L^–1^; gray; *n* = 5–7; **B,D**). K_V_7.1 channel activator ML277-mediated vasorelaxation was not affected by pre-incubation with either BaCl_2_ (100 μmol-L^–1^) or ML133 (20 μmol-L^–1^; gray; *n* = 6–7; **E,F**). In EC denuded vessels, K_V_7.2-5 channel activator ML213 responses were not affected by pre-incubation with either K_IR_2 channel inhibitor BaCl_2_ (100 μmol-L^–1^; gray; *n* = 5; **G**) or ML133 (20 μmol-L^–1^; gray; *n* = 5–6; **H**). All values are expressed as mean + SEM **(A–H)**. A two-way statistical ANOVA with a *post hoc* Bonferroni test was used to generate significance values. Statistical significance is defined as *P < 0.05 **(A–H)**.

In arteries with a functional endothelium, K_IR_2 blockers, BaCl_2_ (100 μmol-L^–1^) and ML133 (20 μmol-L^–1^), significantly impaired relaxations produced by S-1 (Figure 6A, EC_50_ = DMSO, 1.89 ± 0.2 μmol-L^–1^/BaCl_2_, 2.3 ± 0.31 μmol-L^–1^; Figure 6B, EC_50_ = DMSO, 0.52 ± 0.12 μmol-L^–1^/ML133, 3.1 ± 1.5 μmol-L^–1^) and ML213 (Figures 6C,D, EC_50_ = DMSO, 0.9 ± 0.3 μmol/L^–1^/BaCl_2_, 2.2 ± 0.5 μmol/L^–1^/ML133, 2.5 ± 0.25 μmol-L^–1^) when compared to DMSO solvent control (Figures 6A–D). No attenuation of the response to ML277 was observed when pre-incubated with either blocker (Figures 6E,F), consistent with EC removal. Furthermore, in arteries where the endothelium had been removed neither ML133 nor BaCl_2_ had any effect on ML213 mediated relaxations (Figures 6G,H).

### IK_Ca_/SK_Ca_ Inhibitors Had No Impact on K_V_7 Activator Mediated Relaxation

**FIGURE 7:**
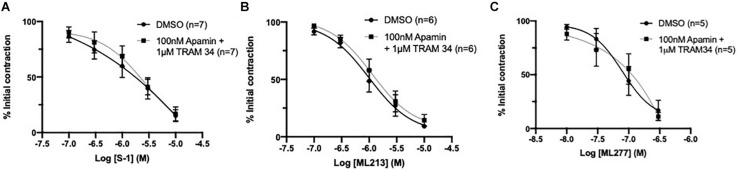
K_V_7 channel activator mediated vasorelaxation was not affected by preincubation with IK_Ca_/SK_Ca_ channel antagonists. Pre-incubation with both IK_Ca_ channel inhibitor TRAM34 (1 μmol-L^–1^) and SK_Ca_ channel inhibitor Apamin (100 nmol-L^–1^) had no effect on S-1, ML213 nor ML277 mediated vasorelaxation (*n* = 5–7; gray, **A–C**). All values are expressed are expressed as mean values ± SEM. A two-way statistical ANOVA with a *post hoc* Bonferroni test was used to generate significance values; no significant values were observed **(A–C)**.

Endothelial IK_Ca_ and SK_Ca_ channels contribute to relaxation responses in rat MA (Crane et al., 2003b). Thus, it is feasible that K_V_7 channels interact with other key endothelial potassium channels; particularly those expressed within microdomains (Sandow et al., 2009). However, consistent with previous reports (Jepps et al., 2016), pre-incubation with a combination of IK_Ca_ inhibitor TRAM-34 (1 μmol-L^–1^; Wulff et al., 2000) and SK_Ca_ inhibitor apamin (100 nmol-L^–1^; Spoerri et al., 1975) had no effect on K_V_7 activator mediated vasorelaxation (Figures 7A–C). These data suggest that the endothelium-dependent increase in potency to the K_V_7 activators involves endothelial K_IR_, but not IK_Ca_ or SK_Ca_ channels.

### K_V_7 Channels Contribute to CCh Evoked Vasorelaxation

The expression of functional K_V_7 channels within ECs begs the question - do they contribute to EC-derived responses? Acetylcholine produces endothelium-dependent relaxation through NO-, EDH- and prostanoid-dependent mechanisms in rat MA (Parsons et al., 1994; Shimokawa et al., 1996; Peredo et al., 1997).

**FIGURE 8:**
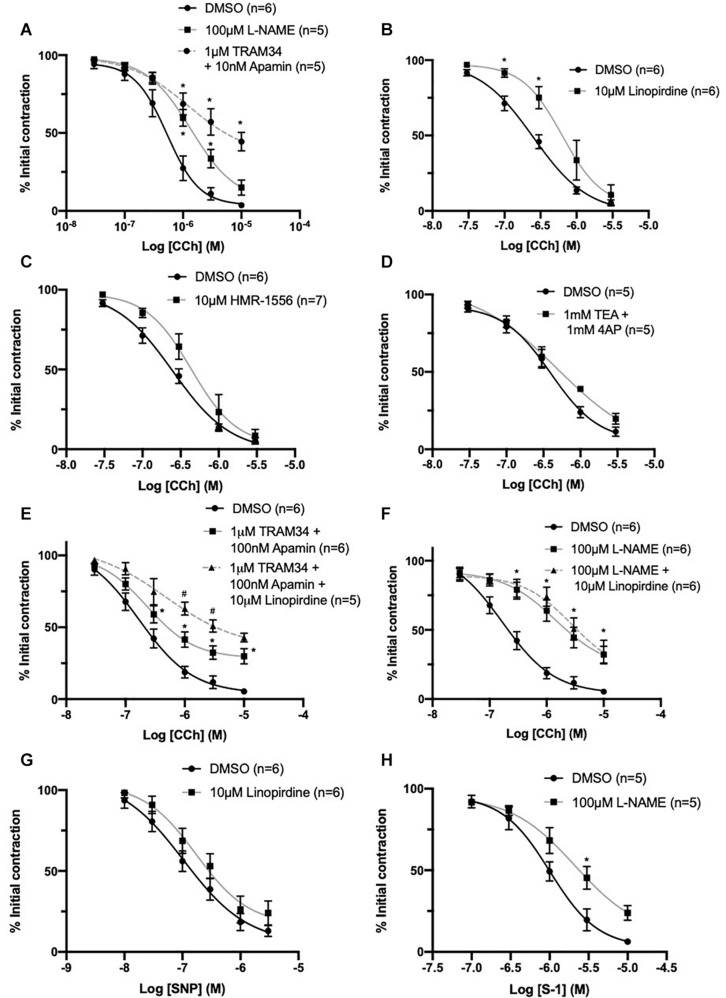
CCh evoked vasorelaxation is mediated through both eNOS and IK_Ca_/SK_Ca_ channels and is partially attenuated by inhibition of K_V_7 channels upstream of NO. Both eNOS inhibitor L-nitroarginine methyl ester (L-NAME; 100 μmol-L^–1^; gray) and IK_Ca_/SK_Ca_ channel inhibitors TRAM-34 (1 μmol-L^–1^)/apamin (100 nmol-L^–1^; red, dashed line) significantly attenuated CCh mediated vasorelaxation (*n* = 5–6; **A**). The pan K_V_7 channel inhibitor linopirdine (10 μmol-L^–1^; gray) significantly attenuated CCh mediated vasorelaxation (*n* = 6; **B**). The K_V_7.1 channel inhibitor HMR-1556 (10 μmol-L^–1^; gray) did not modulate CCh-mediated vasorelaxation (*n* = 6; **C**). A combination of non-specific K^+^ channel inhibitors 4-aminopyridine (4-AP; 1 mmol-L^–1^) and tetraethylammonium (TEA; 1 mmol-L^–1^; gray) did not modulate CCh-mediated vasorelaxation (*n* = 5; **D**). Additive attenuation to CCh-evoked relaxation in the presence of TRAM-34 (1 μmol-L^–1^)/apamin (100 nmol-L^–1^) + linopirdine (10 μmol-L^–1^; gray, dashed line) was observed when compared to vessels pre-incubated in only TRAM34/apamin (gray; *n* = 5–6; **E**). No additive attenuation to CCh evoked vasorelaxation was observed between vessels pre-incubated in L-NAME (100 μmol-L^–1^) + linopirdine (10 μmol-L^–1^; gray, dashed line) when compared with L-NAME (100 μmol-L^–1^; gray; *n* = 5; **F**). SNP-mediated vasorelaxation was no effected by the pan-K_V_7 channel inhibitor linopirdine (10 μmol-L^–1^; gray) when compared to DMSO solvent control (black; **G**). K_V_7.2-5 channel activator S-1 was significantly attenuated by pre-incubation with eNOS inhibitor L-NAME (100 μmol-L^–1^; gray; *n* = 5; **H**). All values are expressed as mean + SEM. A two-way statistical ANOVA with a *post hoc* Bonferroni test was used to generate significance values (*, drug v DMSO solvent control; #=Group B vs. Group C) Statistical significance is defined as ^#/^*P < 0.05 **(A–H)**.

A distinct rightward shift in the sensitivity to vasorelaxation in response to CCh, a synthetic acetylcholine analog, was produced by the eNOS inhibitor L-NAME (100 μmol-L^–1^) when compared to DMSO control (EC_50_ DMSO = 0.59 ± 0.1 μmol-L^–1^; L-NAME = 0.94 ± 0.1 μmol-L^–1^; Figure 8A). A combination IK_Ca_ and SK_Ca_ inhibitors, TRAM-34 (1 μmol-L^–1^) and apamin (100 nmol-L^–1^) respectively, suppress EDH in rat MA, and produced greater attenuation (EC_50_ TRAM34/apamin = 1.5 ± 0.7 μmol-L^–1^; Figure 8A) when compared to L-NAME. Pre-incubating vessels with the pan K_V_7 channel inhibitor linopirdine (10 μmol-L^–1^) significantly attenuated CCh-mediated relaxation when compared to DMSO control (EC_50_ DMSO = 0.2 ± 0.08 μmol-L^–1^; linopirdine = 0.7 ± 0.3 μmol-L^–1^; Figure 8B). In contrast, pre-incubating vessels with either the K_V_7.1 specific inhibitor HMR-1556 (10 μmol-L^–1^) or a combination of non-specific K_V_ channel inhibitors TEA (1 mmol-L^–1^; Choi et al., 1991) and 4-AP (1 mmol-L^–1^; Kurata and Fedida, 2006) had no significant effect on CCh-evoked vasorelaxation (Figures 8C,D).

Additionally, CCh-evoked relaxations were significantly attenuated in vessels pre-incubated in TRAM34/apamin (1 μmol-L^–1^/100 nmol-L^–1^) and linopirdine (10 μmol-L^–1^) compared to vessels only pre-incubated in TRAM34/apamin alone (EC_50_ DMSO = 0.24 ± 0.05 μmol-L^–1^; TRAM34/Apamin = 0.27 ± 0.03 μmol-L^–1^; TRAM34/Apamin + linopirdine = 0.61 ± 0.2 μmol-L^–1^; Figure 8E). In contrast, linopirdine failed to attenuate CCh relaxation in arteries pre-incubated with L-NAME (100 μmol-L^–1^; Figure 8F), thus suggesting K_V_7 contribution to eNOS sensitive proportion of CCh-mediated relaxation.

Furthermore, the present data demonstrates that pre-incubation with linopirdine (10 μmol-L^–1^) has no effect on vasorelaxation produced by the NO-donor SNP (Figure 8G). However, in contrast with previous reports (Jepps et al., 2016), pre-incubation with L-NAME (100 μmol-L^–1^) significantly attenuated K_V_7.2-5 activator mediated vasorelaxation (Figure 8H).

## Discussion

The present study identified *Kcnq1, Kcnq4*, and *Kcnq5* transcripts in EC marker expressing cells and the consequent K_V_7.1, K_V_7.4, and K_V_7.5 protein in isolated and whole mount rat MA ECs/VSMCs. Functionally, the present study demonstrates that the relaxation produced by two structurally different K_V_7.2-5 activators, but not a K_V_7.1 activator, were modulated by the presence of the endothelium and gap junction inhibition. Said relaxation was also sensitive to K_IR_2 inhibition, which was dependent on the presence of ECs, suggestive of a novel functional interaction between K_V_7 and endothelial K_IR_2.x channels. Furthermore, the present data suggest that K_V_7.4/K_V_7.5 channels contribute to the NO-mediated axis of CCh-evoked endothelium-dependent relaxation downstream of eNOS. Thus, K_V_7 channels are expressed in ECs, when pharmacologically upregulated, are functionally coupled to other EC potassium channels in rat MA and contribute to endothelium-derived responses.

### K_V_7 Channel Expression and Function Within ECs

Kv7 channel modulators have considerable impact on arterial tone. In VSMCs, active K_V_7 channels hyperpolarize the membrane potential, decreasing voltage-dependent calcium channel (VDCC) open probability and extracellular calcium influx resulting in relaxation. Within rodent models, the pharmacopeia of K_V_7 channel modulators has revealed K_V_7.4 and 7.5 channels are; (1) key determinants of resting vascular tone via regulation of resting membrane potential; (2) upregulated during cGMP and cAMP/EPAC/PKA-mediated vasodilation; (3) suppressed via PKC-mediated vasoconstriction. Comparatively, no functional role for K_V_7.1 has been identified in arteries (see Barrese et al., 2018b; Byron and Brueggemann, 2018 for review). A caveat of these observations is a lack of differentiation between VSMCs and ECs. However, recently K_V_7 channels were identified in pig coronary artery ECs (Chen et al., 2016), the novel findings presented here expand on these findings and demonstrate K_V_7 transcript and channel expression, functional activity and contribution to EC-derived vasodilatory signaling cascades in rat MA endothelium.

**FIGURE 9:**
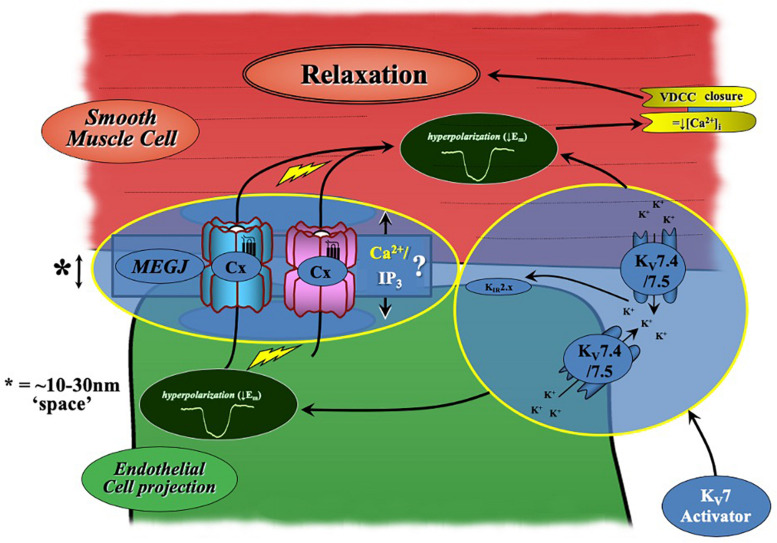
Schematic of K_V_7.2-5 activator-mediated vasorelaxation. K_V_7.2-5 activator-mediated vasorelaxation occurs predominantly via K_V_7.4/7.5 activation within VSMCs. However, our findings suggest a novel contribution of endothelial-K_V_7.4/7.5 channels to K_V_7.2-5 activator mediated responses which augments relaxation. A process which is sensitive to both gap-junction and endothelial-K_IR_2.x inhibition (modified from Senadheera et al., 2012).

In most arteries, the endothelium and smooth muscle are electrochemically linked via MEGJs formed from connexin proteins within heterocellular communicating microdomains present within holes in the IEL (see Sandow et al., 2012 for review). Via these connections, current injection in to ECs passes into VSMCs (Sandow et al., 2002) supporting the presence of such coupling. The present data suggests a potential role for electrochemical heterocellular communication during K_V_7.2-5 activator-mediated vasorelaxation (Figure 9). This conjecture is supported by apparently higher level expression of K_V_7.4/K_V_7.5 in a proportion of IEL holes than that at the EC membrane as well as a significant attenuation of relaxation of pre-constricted arterial tone by two structurally different K_V_7.2-5 activators in the absence of ECs and presence of a (putative) gap junction inhibitor. In contrast, EC removal had no impact on K_V_7.1 activator-mediated vasorelaxation, which was observed in conjunction with reduced expression of *Kcnq1* transcript and K_V_7.1 labeling within ECs compared to VSMCs. Collectively suggesting that only K_V_7.4/K_V_7.5 channels of the K_V_7 sub-family are functionally expressed within MA ECs. An extrapolation of these results indicates a novel role for K_V_7.4/K_V_7.5 channels in the regulation of EC membrane potential within myoendothelial projections. It is plausible, that localized EC K_V_7 channels negate the influx of depolarization from VSMCs into the endothelium, providing tight regulation of EC V_m_ during VSMC contraction. However, further studies would be required to validate this hypothesis.

### A Novel Functional Interaction With Endothelial K_IR_ Channels

K_IR_2.x channels have been characterized in a variety of rat vascular beds including cerebral and coronary arteries (Smith et al., 2008), where their selective inhibition by Ba^2+^ revealed K_IR_ channel amplification of a K^+^ channel activator conductance (Smith et al., 2008). However, there is a degree of conflict regarding the role of K_IR_2.x channels within rat MAs. As above, Ba^2+^ sensitive currents and K_IR_2.x expression has been demonstrated in rat MA ECs (Crane et al., 2003a) which are purported to contribute to K_Ca_ mediated-hyperpolarization during ACh-derived EC-dependent responses (Goto et al., 2004). In contrast, Smith et al. (2008) demonstrated that within 3rd order MAs, ACh-mediated K^+^ conductance-dependent relaxation was insensitive to Ba^2+^, indicating that K_IR_2.x channels do not augment K^+^ conductance in these vessels during receptor-mediated vasodilation.

In the present study, significant attenuation of both S-1 and ML213 K_V_7.2-5 activator-mediated vasorelaxation was found following pre-incubation with two structurally different K_IR_2.x blockers ML133 (Wu et al., 2010) and Ba^2+^ (Hagiwara et al., 1978) when compared to a solvent control. ML133 has been identified via high-throughput and mutagenesis investigation as a novel inhibitor of K_IR_2.1 channels via D172 and I176 residues within the M2 region of K_IR_2.1 (Wang et al., 2011) with an IC_50_ of 1.8 μmol-L^–1^ at pH 7.4, with little to no effect against other K_IR_2.x family members (Wu et al., 2010; Wang et al., 2011). Presently, ML133 is the most selective inhibitor of the K_IR_2 family. In analogous fashion to our endothelium denudation experiments, no effect was seen on the K_V_7.1 activator ML277-mediated vasorelaxation; implying a specific interplay with K_V_7.4 and 7.5 channels. Furthermore, K_IR_2 blockers had no effect on K_V_7.2–7.5 activator-mediated relaxation in EC-denuded arteries, supporting the notion that functional K_IR_2 channels (in the rat MA bed) are restricted to the endothelium (Crane et al., 2003b). The present study suggests that pharmacological activation of endothelial K_V_7.4/7.5 channels also activates an endothelial K_IR_2 channels, increasing K^+^ conductance, which in turn accounts for a proportion of EC augmentation of K_V_7.2-5 activator-mediated vasorelaxation (Figure 9). This may be due to a localized increase in potassium ion concentration in the proximity of the K_IR_2 channels or an alternative modulation. However, based on the findings described by Goto et al. (2004) and Smith et al. (2008), the collective data suggest that this phenomenon is dependent on the branch order of MA. Furthermore, it remains unclear if this occurs during receptor mediated signaling, or if it is only present during pharmacological activation of endothelial K_V_7 channels.

A primary concern for identification of novel functional interactions between ion channels using pharmacological tools is potential off-target effects. However, K_V_7 activator-mediated relaxation in vessels pre-incubated in 10 μmol-L^–1^ linopirdine was abolished. If S-1 or ML213 were activating non-K_V_ channels such as K_IR_, a degree of relaxation would still be observed in the presence of linopirdine. The present findings therefore suggest that both S-1 and ML213 work exclusively via K_V_7 channels and that attenuation of their response within rat MA occurs via a novel functional coupling of K_V_7.4/7.5 and K_IR_2 within ECs.

### The Contribution of K_V_7 Channels to CCh Evoked Relaxations Within Rat MA

Our findings demonstrate that both NO- and EDH-dependent signaling contributes to CCh-mediated vasodilation, though the main contributor to endothelial-dependent vasodilation in 2nd order MA appears to be EDH, consistent with previous studies (Shimokawa et al., 1996). In light of the significant impact of K_V_7 inhibition on CCh-mediated vasorelaxation during the suppression of EDH, the present study suggests that K_V_7 channels contribute to the eNOS sensitive axis of CCh-evoked relaxation within rat MA.

However, in a similar manner to rat renal artery (Stott et al., 2015), rat MA K_V_7 channels do not represent downstream targets of NO signaling as K_V_7 inhibition does not impair NO-donor SNP-mediated relaxation. However, eNOS inhibition does impair relaxation to a K_V_7 activator, potentially implying K_V_7 channel involvement in the production or release of NO in response to CCh. Interestingly, this appears to be a vascular bed specific phenomenon, as L-NAME has no effect on K_V_7 activator-mediated relaxation within rat penile artery (Jepps et al., 2016).

### Limitations

Ideally, direct functional evidence for a functional role of K_v_7 channels in ECs would be provided by either single cell electrophysiology of sharp microelectrode impalement in whole arteries. However, this was not possible within the constraints of this study. Instead, we inferred a functional role for EC K_v_7 channels using a reductive approach, i.e., comparison of functional responses without or with EC ablation. These studies implicate K_v_7 channels as functional component of the EC physiology that merit consideration in future studies.

## Conclusion

In conclusion, the present data reveal that mesenteric ECs express K_V_7.4/K_V_7.5 channels and their presence boosts K_V_7 activator-mediated relaxation. Furthermore, the data indicated a novel functional interaction with endothelial K_IR_2 channels and support the proposition that endothelial K_V_7 channels contribute to endogenous endothelium-derived responses. These findings highlight the complex nature of the vascular response to K_V_7 channel upregulation and emphasize the importance of these channels to vascular signaling. The present data are consistent with K_V_7 channels representing a novel therapeutic target in endothelial dysfunction.

## Data Availability Statement

The original contributions presented in the study are included in the article/Supplementary Material, further inquiries can be directed to the corresponding author/s.

## Ethics Statement

The animal study was reviewed and approved by UNSW Animal Ethics and Experimentation Committee (AEEC #18/86B). For investigations performed at St George’s University, London, investigators strictly adhered to the Animal (Scientific Procedures) Act 1986.

## Author Contributions

SB, SS, GM-P, and JS performed the research. IG and JS designed the research study. SS contributed essential reagents or tools. SB, SS, and GM-P analyzed the data. SB and IG wrote the manuscript. All the authors contributed to the article and approved the submitted version.

## Conflict of Interest

The authors declare that the research was conducted in the absence of any commercial or financial relationships that could be construed as a potential conflict of interest.

## Abbreviations

4-AP: 4-aminopyridine
Acta2: α-smooth muscle actin-2
CCh: carbachol
cGMP: cyclic guanosine monophosphate
Cq: quantification cycle
CYC1: cytochrome C1
EC: endothelial cell
EDH: endothelium derived hyperpolarization
GAPD: glyceraldehyde-3-phosphate dehydrogenase
eNOS: endothelial nitric oxide synthase
IK_Ca_: intermediate conductance calcium-activated potassium channel
IEL: internal elastic lamina
K_IR_: inwardly rectifying potassium channel
K_V_: voltage-gated potassium channel
L-NAME: L-nitroarginine methyl ester
MA: mesenteric artery
MO: methoxamine
Myh11: myosin heavy chain 11
NO: nitric oxide
PECAM-1: platelet endothelial cell adhesion molecule-1
RT-qPCR: reverse-transcription quantitative polymerase chain reaction
SEM: standard error of the mean
SK_Ca_: small conductance calcium-activated potassium channel
SNP: *S*-nitroprusside
TEA: tetraethylammonium
VSMC: vascular smooth muscle cell
vWBF: von-Willebrand factor.
