# Supplementary material for: KV7 Channel Expression and Function Within Rat Mesenteric Endothelial Cells
Source: Front Physiol. 2020 Dec 7;11:598779. doi: 10.3389/fphys.2020.598779 (PMC7750541; doi:10.3389/fphys.2020.598779)
Supplement: Supplementary file 1 [file Presentation_1.pptx]

## Slide 1
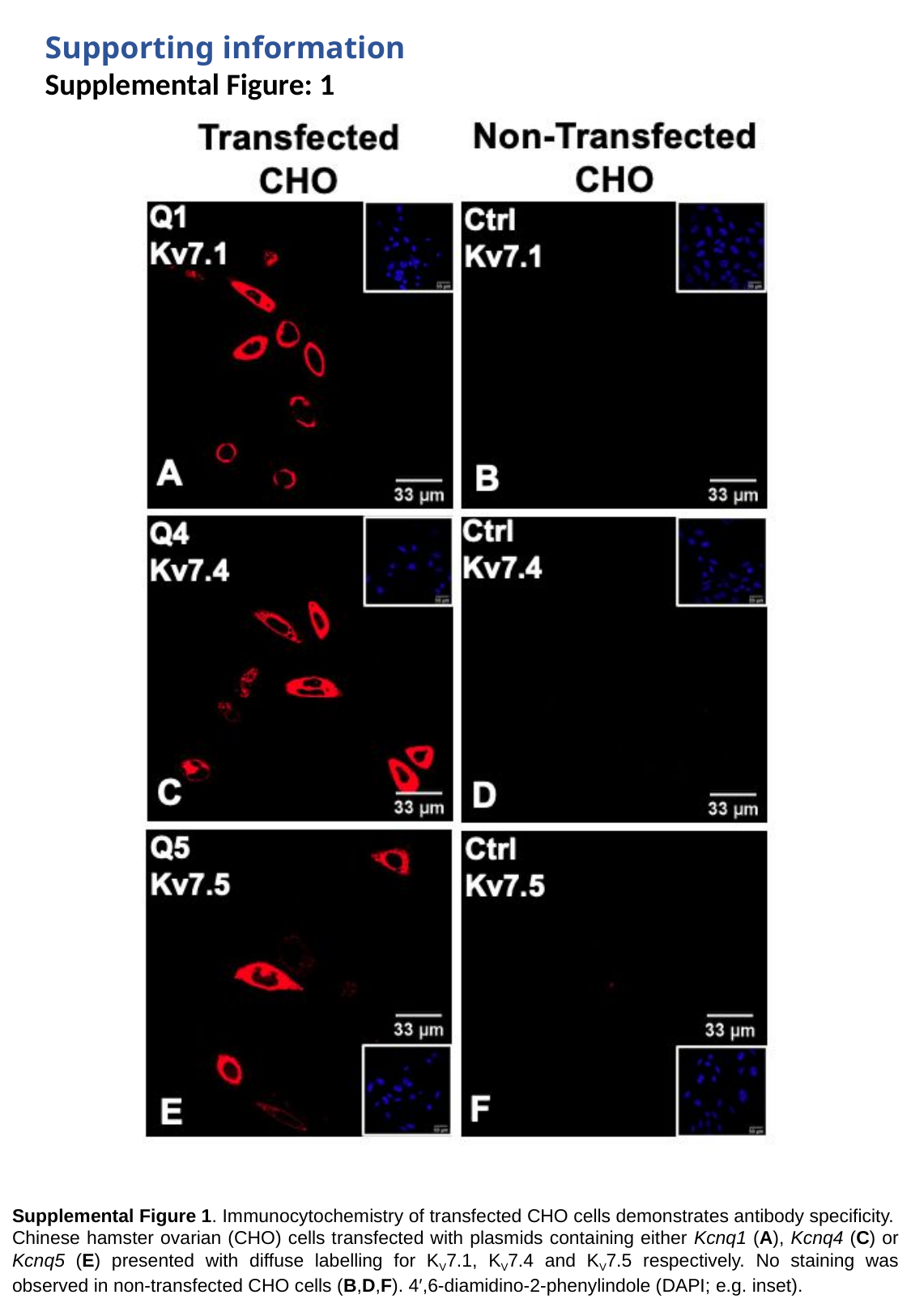

Supporting information
Supplemental Figure: 1
Supplemental Figure 1. Immunocytochemistry of transfected CHO cells demonstrates antibody specificity.
Chinese hamster ovarian (CHO) cells transfected with plasmids containing either Kcnq1 (A), Kcnq4 (C) or Kcnq5 (E) presented with diffuse labelling for KV7.1, KV7.4 and KV7.5 respectively. No staining was observed in non-transfected CHO cells (B,D,F). 4′,6-diamidino-2-phenylindole (DAPI; e.g. inset).

## Slide 2
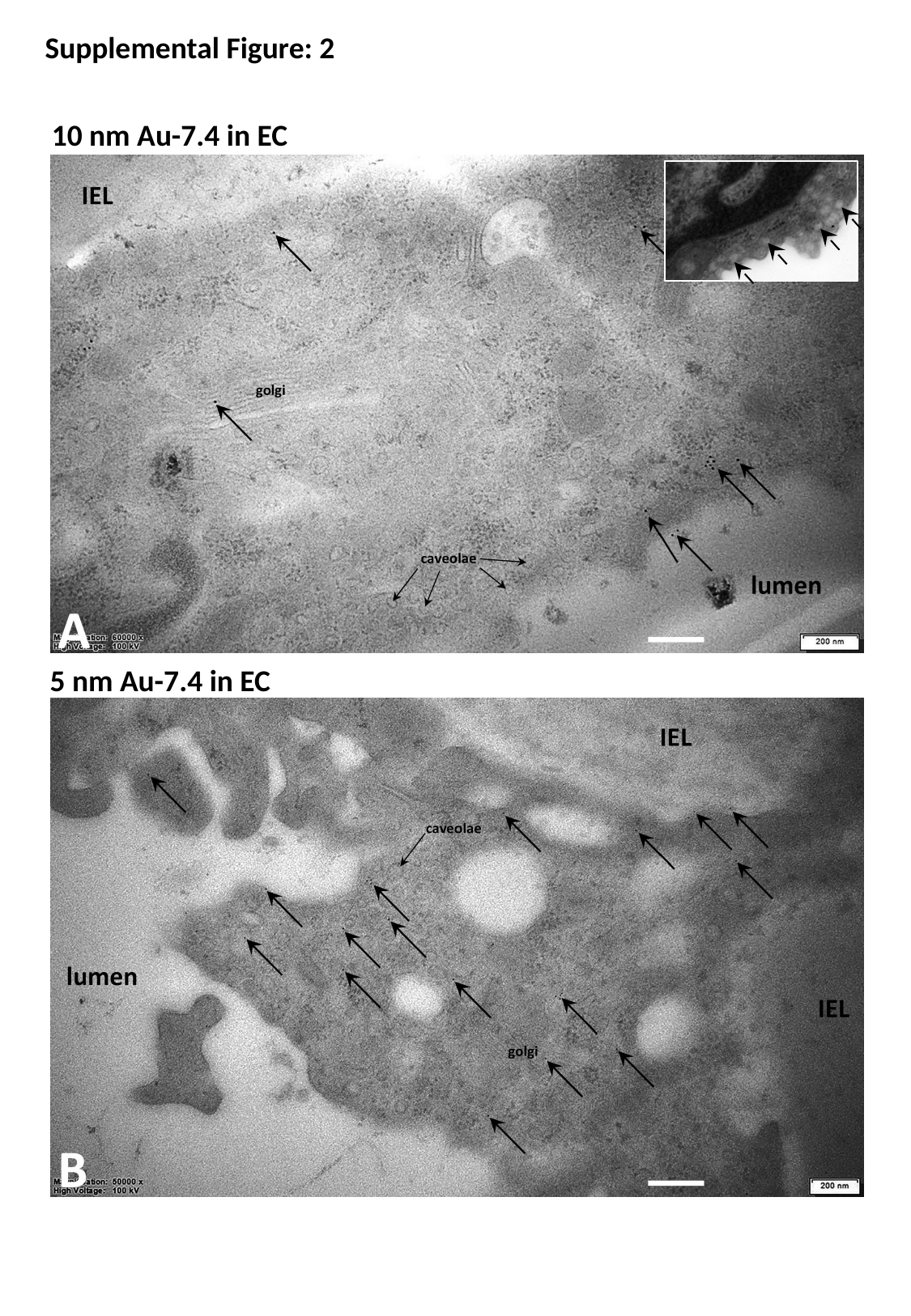

Supplemental Figure: 2
10 nm Au-7.4 in EC
Supplemental data 2.
5 nm Au-7.4 in EC

## Slide 3
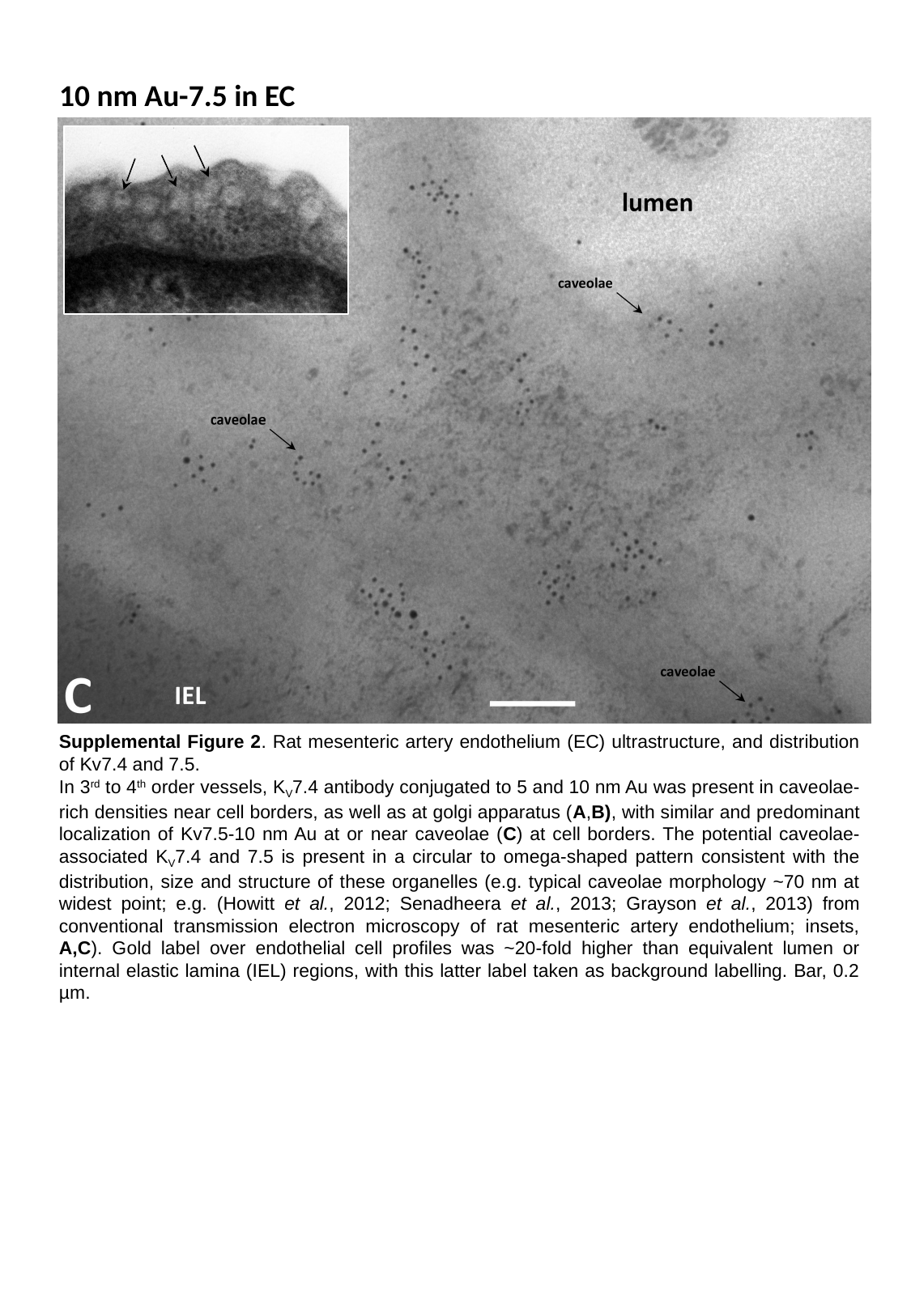

10 nm Au-7.5 in EC
Supplemental Figure 2. Rat mesenteric artery endothelium (EC) ultrastructure, and distribution of Kv7.4 and 7.5.
In 3rd to 4th order vessels, KV7.4 antibody conjugated to 5 and 10 nm Au was present in caveolae-rich densities near cell borders, as well as at golgi apparatus (A,B), with similar and predominant localization of Kv7.5-10 nm Au at or near caveolae (C) at cell borders. The potential caveolae-associated KV7.4 and 7.5 is present in a circular to omega-shaped pattern consistent with the distribution, size and structure of these organelles (e.g. typical caveolae morphology ~70 nm at widest point; e.g. (Howitt et al., 2012; Senadheera et al., 2013; Grayson et al., 2013) from conventional transmission electron microscopy of rat mesenteric artery endothelium; insets, A,C). Gold label over endothelial cell profiles was ~20-fold higher than equivalent lumen or internal elastic lamina (IEL) regions, with this latter label taken as background labelling. Bar, 0.2 µm.
